# Mechanistic insights into the GEF activity of the human MON1A/CCZ1/C18orf8 complex

**DOI:** 10.1093/procel/pwaf018

**Published:** 2025-02-28

**Authors:** Yubin Tang, Yaoyao Han, Zhenpeng Guo, Ying Li, Xinyu Gong, Yuchao Zhang, Haobo Liu, Xindi Zhou, Daichao Xu, Yixiao Zhang, Lifeng Pan

**Affiliations:** State Key Laboratory of Chemical Biology, Shanghai Institute of Organic Chemistry, University of Chinese Academy of Sciences, Chinese Academy of Sciences, Shanghai 200032, China; Interdisciplinary Research Center on Biology and Chemistry, Shanghai Institute of Organic Chemistry, Chinese Academy of Sciences, Shanghai 200032, China; State Key Laboratory of Chemical Biology, Shanghai Institute of Organic Chemistry, University of Chinese Academy of Sciences, Chinese Academy of Sciences, Shanghai 200032, China; State Key Laboratory of Chemical Biology, Shanghai Institute of Organic Chemistry, University of Chinese Academy of Sciences, Chinese Academy of Sciences, Shanghai 200032, China; State Key Laboratory of Chemical Biology, Shanghai Institute of Organic Chemistry, University of Chinese Academy of Sciences, Chinese Academy of Sciences, Shanghai 200032, China; State Key Laboratory of Chemical Biology, Shanghai Institute of Organic Chemistry, University of Chinese Academy of Sciences, Chinese Academy of Sciences, Shanghai 200032, China; State Key Laboratory of Chemical Biology, Shanghai Institute of Organic Chemistry, University of Chinese Academy of Sciences, Chinese Academy of Sciences, Shanghai 200032, China; State Key Laboratory of Chemical Biology, Shanghai Institute of Organic Chemistry, University of Chinese Academy of Sciences, Chinese Academy of Sciences, Shanghai 200032, China; State Key Laboratory of Chemical Biology, Shanghai Institute of Organic Chemistry, University of Chinese Academy of Sciences, Chinese Academy of Sciences, Shanghai 200032, China; Interdisciplinary Research Center on Biology and Chemistry, Shanghai Institute of Organic Chemistry, Chinese Academy of Sciences, Shanghai 200032, China; State Key Laboratory of Chemical Biology, Shanghai Institute of Organic Chemistry, University of Chinese Academy of Sciences, Chinese Academy of Sciences, Shanghai 200032, China; Interdisciplinary Research Center on Biology and Chemistry, Shanghai Institute of Organic Chemistry, Chinese Academy of Sciences, Shanghai 200032, China; State Key Laboratory of Chemical Biology, Shanghai Institute of Organic Chemistry, University of Chinese Academy of Sciences, Chinese Academy of Sciences, Shanghai 200032, China; School of Chemistry and Materials Science, Hangzhou Institute for Advanced Study, University of Chinese Academy of Sciences, Hangzhou 310024, China

**Dear Editor,**

Rab7A (Ras-related protein Rab-7a), a member of the Rab family of small GTPases, has been demonstrated to be a crucial regulator of mammalian autophagic and endosomal pathways (Gutierrez et al., 2004; Yamano et al., 2018). During mammalian autophagy, Rab7A can facilitate the maturation of autophagosomes and their fusion with lysosomes through its interactions with relevant regulatory proteins and binding partners (Chua et al., 2011; Gutierrez et al., 2004; Stroupe, 2018; Vaites et al., 2018). Particularly, the GDP (guanosine-5'-diphosphate) -bound inactive Rab7A is reported to be recruited to autophagosomes by its GEF (guanine nucleotide exchange factor) protein, the MON1/CCZ1/C18orf8 complex (Fig. 1A), which can specifically convert Rab7A to its GTP (guanosine-5'-triphosphate)-bound active state (Nordmann et al., 2010). The precise spatiotemporal regulation of the activity of Rab7A is critical for proper autophagic flux in mammals. Recently, two Cryo-EM (cryo-electron microscopy) structures of the Mon1/Ccz1/Bulli complex from *Drosophila* were reported (Herrmann et al., 2023; Yong et al., 2023). However, due to the lack of complex structures, how the mammalian MON1 (monensin sensitivity 1)/CCZ1 (calcium caffeine zinc sensitivity 1)/C18orf8 (chromosome 18 open reading frame 8) complex specifically recognizes the GDP-bound inactive Rab7A and achieves its specific GEF activity for Rab7A remains unclear.

**Figure 1.**
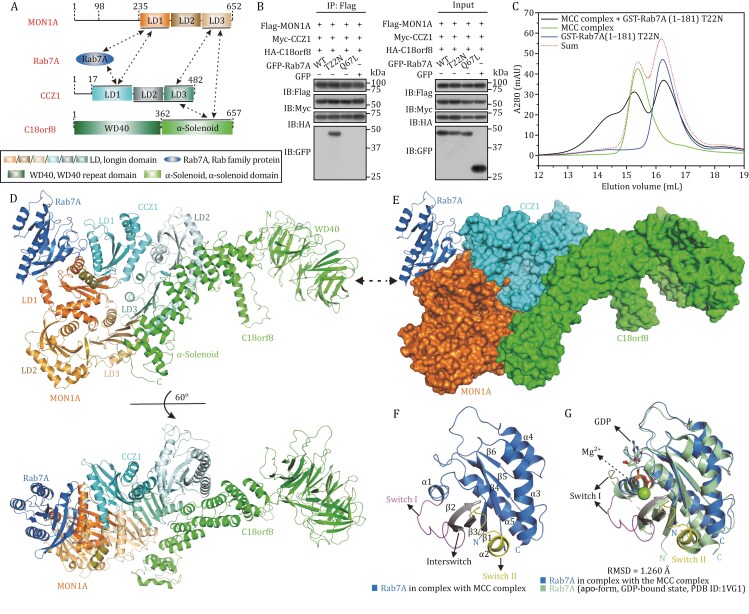
**Biochemical and structural characterizations of the interaction between the MON1A/CCZ1/C18orf8 complex and Rab7A.** (A) A schematic diagram showing the domain organizations of human MON1A, CCZ1, C18orf8, and Rab7A. In this drawing, the MON1A/CCZ1, MON1A/C18orf8, CCZ1/C18orf8, MON1A/Rab7A, and CCZ1/Rab7A interactions are further highlighted and indicated by two-way arrows. (B) Co-immunoprecipitation assays showing that the GDP-bound inactive Rab7A T22N mutant strongly interacts with the MON1A/CCZ1/C18orf8 complex in cells, while the wild-type Rab7A and the GTP-bound active Q67L mutant of Rab7A fail to interact with the MON1A/CCZ1/C18orf8 complex. “WT” means the wild-type Rab7A. “IB” stands for immunoblotting. (C) SEC-based analysis of the interaction of the MON1A/CCZ1/C18orf8 complex with the T22N mutant of Rab7A (1–181). A280, the UV absorbance at 280 nm. (D) Ribbon diagram showing the overall structure of the MON1A/CCZ1/C18orf8/Rab7A complex. (E) The ribbon-surface representation showing the overall architecture of the MON1A/CCZ1/C18orf8/Rab7A complex with the same orientation as in panel (D). In this drawing, the MON1A/CCZ1/C18orf8 complex is shown in the surface representation, and Rab7A is shown in the ribbon model. (F) Ribbon diagram showing the structure of the Rab7A molecule in the MON1A/CCZ1/C18orf8/Rab7A complex. The Switch I, Interswitch, and Switch II of bound Rab7A are further indicated and colored in magenta, gray, and yellow, respectively. (G) Ribbon diagram showing the structural comparison of the Rab7A in the MON1A/CCZ1/C18orf8/Rab7A complex with the *apo*-form GDP-bound Rab7A (PDB ID: 1VG1).

To uncover how the MON1A/CCZ1/C18orf8 complex specifically recognizes Rab7A and conducts its GEF activity, we first performed relevant Co-IP (co-immunoprecipitation) assays and confirmed that only the GDP-bound inactive T22N mutant of Rab7A but not the wild-type or the GTP-bound active Q67L mutant of Rab7A can associate with the MON1A/CCZ1/C18orf8 complex in cells (Fig. 1B). To facilitate following biochemical and structural characterizations of the interaction between the MON1A/CCZ1/C18orf8 complex and Rab7A, we deleted the C-terminal membrane-associating region (residues 182–207) of Rab7A, and constructed a short Rab7A (1–181) fragment (Fig. S1). Subsequently, we purified the Q67L and T22N mutants of Rab7A (1–181) from *E*. *coil* cells, and conducted size exclusion chromatography (SEC)-based analyses to evaluate their interactions with the MON1A/CCZ1/C18orf8 complex. Based on our SEC results, the MON1A/CCZ1/C18orf8 complex can readily interact with the GDP-bound Rab7A (1–181) T22N but not the GTP-bound Rab7A (1–181) Q67L (Figs. 1C and S2A). Subsequently, we purified the MON1A/CCZ1/C18orf8/Rab7A (1–181) T22N quaternary complex, and further MALS (Multi Angle Light Scattering)-based measurement revealed that it forms a 1:1:1:1 stoichiometric hetero-tetramer in solution (Fig. S2B).

Then, based on previous structural studies of the fly Mon1/Ccz1/Bulli complex together with our relevant sequence alignment and Co-IP results (Fig. S3), we used the truncated MON1A (235–652) fragment for assembling the MON1A/CCZ1/C18orf8 complex, and subsequently determined the Cryo-EM structure of the MON1A (235–652)/CCZ1 (1–482)/C18orf8 (1–657)/Rab7A (1–181) T22N complex to a 3.41 Å resolution (Figs. S4–S7; Table S1), revealing a unique architecture of the MON1A/CCZ1/C18orf8 complex bound with one Rab7A (1–181) T22N (Fig. 1D and 1E). In the complex structure, both MON1A and CCZ1 are assembled in a triangular manner by three LD (Longin) domains, and directly interact with the C-terminal α-solenoid domain of C18orf8 forming the MON1A/CCZ1/C18orf8 complex (Fig. 1D and 1E). Meanwhile, Rab7A adopts a typical Rab GTPase fold, and packs against a solvent-exposed surface co-assembled by the two LD1 domains of MON1A and CCZ1 to form a triangular-like architecture (Fig. 1D and 1E). Intriguingly, the GDP molecule and the related Mg^2+^ co-factor of Rab7A T22N are missing in the structure (Fig. 1F), and the overall configurations of the Switch I and Switch II regions of Rab7A in the MON1A/CCZ1/C18orf8/Rab7A complex are significantly different from that of the *apo*-form GDP-bound Rab7A (PDB ID (Protein data bank ID): 1VG1), the *apo*-form GTP-bound Rab7A (PDB ID: 1T91), or the GTP-bound Rab7A in the Rubicon/Rab7 complex (PDB ID: 6WCW) (Figs. 1G and S8).

Further structural analyses of the binding interface of Rab7A (1–181) T22N with the MON1A/CCZ1/C18orf8 complex revealed that the specific association of Rab7A with the two LD1 domains of MON1A and CCZ1 is mainly mediated by both hydrophobic and hydrophilic interactions (Figs. 2A, 2B and S9). Specifically, the hydrophobic side chain groups of Y37, I41 from the Switch I of Rab7A, and F45, W62 from the Interswitch as well as L73 from the Switch II of Rab7A occupy the hydrophobic groove formed by the V281, V283, A284, L285, and F288 residues from the MON1A LD1 domain and the L64, A67, and F71 residues from the CCZ1 LD1 domain (Fig. 2A and 2B). Meanwhile, the hydrophobic side chain of F33 within Switch I of Rab7A forms a hydrophobic contact with the A274 residue from the MON1A LD1 domain (Fig. 2A and 2B). Furthermore, the side chains of K10, N22, T40, T58, Q60, Q67, E68, R79, and D82 together with the main chain carbonyl group of L8 from Rab7A interact with the A261, E273, G262, E260, N358, and D360 residues from MON1A LD1 and the E38, E66, and N56 residues from CCZ1 LD1, forming 10 specific hydrogen bonds (Fig. 2B). In parallel, the positively charged K31 and R69 from Rab7A couple with the negatively charged E238 from MON1A LD1 and E41 from CCZ1 LD1 to form two pairs of salt bridges, respectively (Figs. 2B and S9). In line with our structural data, further SEC-based analyses confirmed that the Y37Q or F45Q mutation of Rab7A (1–181) T22N, can essentially abolish the interaction between Rab7A (1–181) T22N and the MON1A/CCZ1/C18orf8 complex (Fig. S10A and S10B). Consistently, further Co-IP assays demonstrated that point mutations of key residues involved in the binding interface between the MON1A/CCZ1/C18orf8 complex and Rab7A, completely disrupt the specific interaction between Rab7A T22N and the MON1A/CCZ1/C18orf8 complex (Fig. S10C).

**Figure 2.**
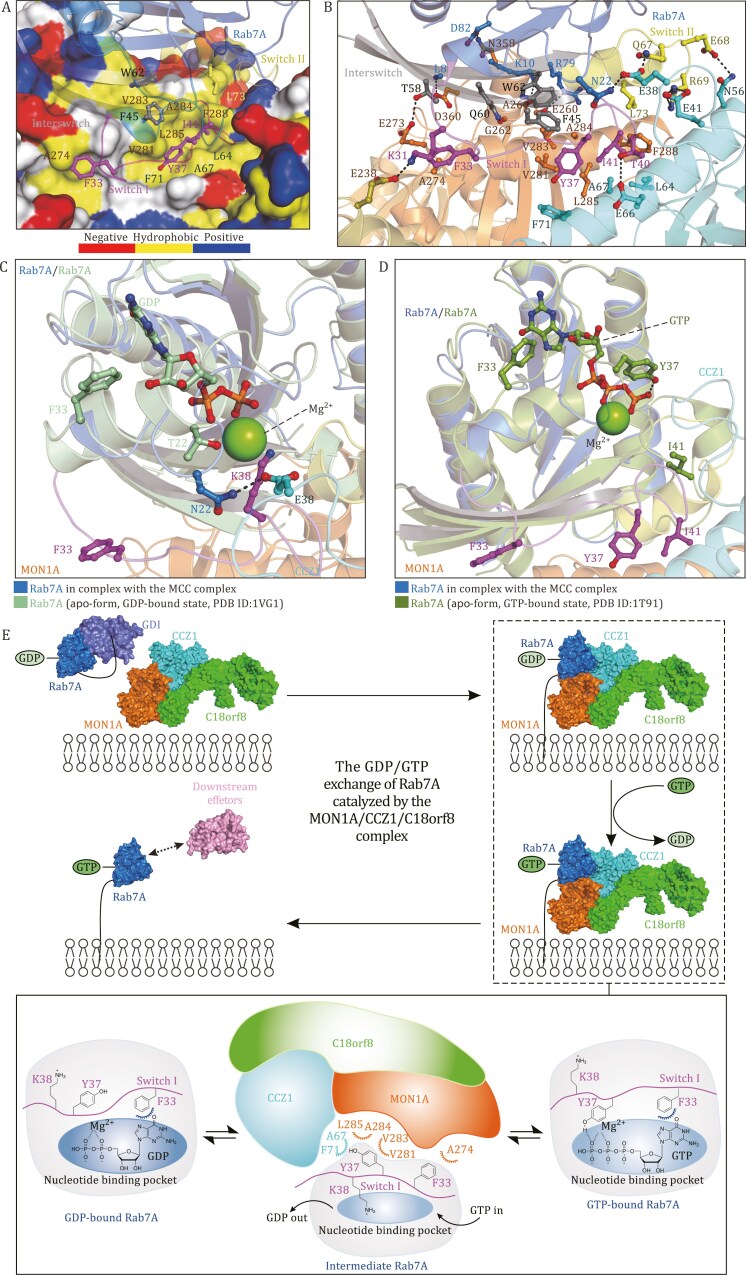
**The binding and GEF mechanisms of the MON1A/CCZ1/C18orf8 complex for Rab7A.** (A) The combined surface representation and ribbon-stick model showing the hydrophobic binding surface between the MON1A/CCZ1/C18orf8 complex and Rab7A. In this representation, Rab7A is displayed in the ribbon-stick model, and the MON1A/CCZ1/C18orf8 complex is shown in the surface model colored by different amino acid types. (B) The ribbon-stick representation showing the detailed binding interface of the MON1A/CCZ1/C18orf8 complex with Rab7A. In this drawing, the side chains of the key residues involved in the binding interface are shown in stick-ball mode, and the related hydrogen bonds are shown as dotted lines. (C and D) The ribbon-stick representation showing the detailed structure comparison of Rab7A in the MON1A/CCZ1/C18orf8/Rab7A complex with the *apo*-form GDP-bound inactive Rab7A (PDB ID: 1VG1) (C), or the *apo*-form GTP-bound active Rab7A (PDB ID: 1T91) (D). In these drawings, the side chains of the key residues as well as the bound GDP or GTP molecules in Rab7A are shown in the stick-ball mode, and the related hydrogen bonds are shown as dotted lines. Notably, the side chain of the Rab7A K38 residue in the MON1A/CCZ1/C18orf8/Rab7A complex inserts into the nucleotide-binding pocket of Rab7A, and occupies the position of the Mg^2+^ ion in the *apo*-form GDP-bound Rab7A. While, the side chain of Y37 in the GTP-bound Rab7A forms a strong hydrogen bond with the γ-phosphate group of GTP in the *apo*-form GTP-bound Rab7A. (E) A proposed model for describing the GDP/GTP exchange of Rab7A catalyzed by the MON1A/CCZ1/C18orf8 complex in mammals. In this model, MON1A is shown in orange, CCZ1 is shown in cyan, C18orf8 is shown in green, Rab7A is shown in marine, the related GDI and downstream effectors are shown in purple and pink, respectively. The Switch I of the bound Rab7A is colored in magenta.

Careful structural comparison analyses revealed that the most prominent distinction between Rab7A T22N in the MON1A/CCZ1/C18orf8/Rab7A complex and the *apo*-form GDP-bound Rab7A (PDB ID: 1VG1) lies in the conformation of the Switch I of Rab7A (Figs. 1G and 2C). Particularly, in the *apo*-form GDP-bound Rab7A, the F33 residue within the Switch I is upward-facing, and is approaching the nucleotide-binding pocket by forming an edge-to-face π-π interaction with the guanine nucleotide (Fig. 2C). Meanwhile, the hydroxyl group of T22 is coordinated with the Mg^2+^ ion (Fig. 2C). In contrast, the Switch I of Rab7A T22N in the MON1A/CCZ1/C18orf8/Rab7A complex flips down and directly binds to MON1A and CCZ1 mainly via the hydrophobic interactions of the F33, Y37, I41 residues of Rab7A T22N with the relevant hydrophobic residues of MON1A and CCZ1, thereby destabilizing the nucleotide binding of Rab7A T22N (Fig. 2C). Moreover, the positively charged side chain of Rab7A K38 inserts into the nucleotide-binding pocket of Rab7A, and occupies the position of Mg^2+^ in the GDP-bound Rab7A (Fig. 2C). Notably, due to the loss of the Mg^2+^ ion, the N22 residue of Rab7A T22N engages with CCZ1 E38 rather than the bound Mg^2+^ ion (Fig. 2C). Altogether, it is clear that when binding to the GDP-bound Rab7A, the MON1A/CCZ1/C18orf8 complex can induce a large structural rearrangement of the Switch I of Rab7A to destabilize the GDP-binding of Rab7A, facilitating the dissociation of GDP and the subsequent loading of GTP. Interestingly, our structural comparison analysis of Rab7A T22N in the MON1A/CCZ1/C18orf8/Rab7A complex with the *apo*-form GTP-bound Rab7A (PDB ID: 1T91) revealed that the key F33 and Y37 residues of Rab7A directly interact with GTP in the active Rab7A (Fig. 2D). Specifically, the side chain of Y37 in the GTP-bound Rab7A forms a strong hydrogen bond with the γ-phosphate group of GTP in the active Rab7A (Fig. 2D). Since the γ-phosphate group of GTP is absence in GDP, the key Y37 residue in the GDP-bound state but not the GTP-bound state of Rab7A is accessible for binding to the MON1A/CCZ1/C18orf8 complex. Apparently, once GTP is loaded into Rab7A during the catalytic process mediated by the MON1A/CCZ1/C18orf8 complex, GTP can directly compete with the MON1A/CCZ1/C18orf8 complex for binding to the Y37 and F33 residues of Rab7A, ultimately resulting in the release of the GTP-bound active Rab7A from the MON1A/CCZ1/C18orf8 complex. In contrast to that of the GDP-bound Rab7A, due to the presence of a strong hydrogen bond between GTP and the side chain of Rab7A Y37, the Switch I region of the GTP-bound wild-type Rab7A or Rab7A Q67L mutant is not accessible for the MON1A/CCZ1/C18orf8 complex (Figs. 2D and S11). Therefore, only the GDP-bound Rab7 but not the GTP-bound Rab7A can bind to the MON1A/CCZ1/C18orf8 complex.

Notably, the overall binding mode of Rab7A with the human MON1A/CCZ1/C18orf8 complex is similar to that of the *Chaetomium thermophile* (*Ct*) Ypt7 with the *Ct*Mon1 LD1/Ccz1 LD1 complex (PDB ID: 5LDD) (Fig. S12). Importantly, similar conformational changes of the Switch I regions of human Rab7A and *Ct*Ypt7 (*Chaetomium thermophile* Ypt/Rab-type GTPase ypt7) upon binding to the human MON1A/CCZ1/C18orf8 complex or the *Ct*Mon1 LD1/Ccz1 LD1 complex were observed (Fig. S12), revealing a conserved mechanism shared by the human MON1A/CCZ1/C18orf8 complex and the *Ct*Mon1/Ccz1 complex to conduct their GEF activities (Kiontke et al., 2017).

The activation of Rab7A by the MON1A/CCZ1/C18orf8 complex is a key step in the maturation of autophagosome, and is subject to the precise spatiotemporal control of the localization of the MON1A/CCZ1/C18orf8 complex to autophagosome. However, currently we have no idea on the mechanism underpinning the recruitment of the MON1A/CCZ1/C18orf8 complex to autophagosome membrane. Thus, more studies are required to elucidate the precise mechanism governing the recruitment of the MON1A/CCZ1/C18orf8 complex to autophagosome during mammalian autophagy processes in the future.

In all, we determined the Cryo-EM structure of the human MON1A/CCZ1/C18orf8/Rab7A T22N complex, and uncovered that the binding of the MON1A/CCZ1/C18orf8 complex to Rab7A can induce large conformational changes of the Switch I region of Rab7A to promote the GDP/GTP exchange of Rab7A, thereby shedding light on the working mechanism of the MON1A/CCZ1/C18orf8 complex to conduct its GEF activity for Rab7A. Finally, we proposed a model to depict the GDP/GTP exchange of Rab7A catalyzed by the MON1A/CCZ1/C18orf8 complex in mammals (Fig. 2E). In this model, the MON1A/CCZ1/C18orf8 complex is first recruited to specific membrane structures, such as the endosome membrane and autophagosome (Fig. 2E). Meanwhile, the GDP-bound inactive Rab7A is kept soluble in the cytoplasm by binding to the chaperone-like GDP dissociation inhibitor (Fig. 2E). Then, the GDP-bound inactive Rab7A is recognized by the MON1A/CCZ1 sub-complex within the MON1A/CCZ1/C18orf8 complex, and recruited to relevant membrane structure, where the MON1A/CCZ1/C18orf8 complex resides (Fig. 2E). Subsequently, the binding of the MON1A/CCZ1/C18orf8 complex to the inactive Rab7A induces large structural rearrangements of many residues within the Switch I region of Rab7A, especially the key F33, Y37, and K38 residues (Fig. 2E), thereby promoting the GDP/GTP exchange of Rab7A in a way as uncovered in this study. Finally, the GTP-bound active Rab7A disassociates from the MON1A/CCZ1/C18orf8 complex, and is specifically recognized by relevant Rab7A effectors to trigger different downstream cellular processes (Fig. 2E).

## Supplementary Material

pwaf018_suppl_Supplementary_Figures_S1-S12_Tables_S1

## Footnotes

We thank Prof. Junying Yuan from Interdisciplinary Research Center on Biology and Chemistry, CAS, China for providing the HEK293T cell lines, and the Cryo-Electron Microscopy center at the Interdisciplinary Research Center on Biology and Chemistry, Shanghai Institute of Organic Chemistry for helping with data collection.

This work was supported by grants from the Strategic Priority Research Program of the Chinese Academy of Sciences (XDB1060000), the National Basic Research Program of China (2022YFC2303102), the National Natural Science Foundation of China (92253301, 32071219, 21822705), the CAS Youth Interdisciplinary Team (JCTD-2022-10) (to L.P.), the STI2030-Major Project (2022ZD0207400) (to Y.Z.), the Shanghai Key Laboratory of Aging Studies (19DZ2260400) (to Y.Z.), the Basic Research Pioneer Project by the Science and Technology Commission of Shanghai Municipality (STCSM).

The authors declared that there is no conflict of interest. All authors give their consent for participation and publishing.

The following Cryo-EM map and atomic coordinate have been deposited to EM Data Bank and Protein Data Bank: MCCR-complex (EMD-62697, PDB-9L0D). All data needed to evaluate the conclusions in the paper are present in the paper and/or the Supplementary Materials.

Y.T., Y.H., Yixiao Zhang, and L.P. designed research; Y.T., Y.H., Z.G., and Y.L. performed research; X.G., Yuchao Zhang, X.Z., and D.X. provided relevant research reagents; Y.T., Y.H., Yixiao Zhang, and L.P. analyzed data; Y.T., Y.H., Yixiao Zhang, and L.P. wrote the paper.

AlphaFold2 was used to generate the structure models of the human MON1A, CCZ1, C18orf8, and Rab7A.
