# Supplementary material for: Mechanistic insights into the GEF activity of the human MON1A/CCZ1/C18orf8 complex
Source: Protein Cell. 2025 Feb 28;16(8):739–44. doi: 10.1093/procel/pwaf018 (PMC12342132; doi:10.1093/procel/pwaf018)
Supplement: pwaf018_suppl_Supplementary_Figures_S1-S12_Tables_S1 [file pwaf018_suppl_supplementary_figures_s1-s12_tables_s1.pdf]

Supplementary Materials for

Mechanistic insights into the GEF activity of the human MON1A/CCZ1/C18orf8 complex

Yubin Tang, Yaoyao Han, Zhenpeng Guo, Ying Li, Xinyu Gong, Yuchao Zhang, Haobo Liu, Xindi Zhou, Daichao Xu, Yixiao Zhang*, and Lifeng Pan*

Materials and methods

Plasmid construction

Different DNA fragments, which encoded human MON1A, CCZ1, C18orf8, Rab7A and other related DNA fragments, were amplified using the polymerase chain reaction (PCR) method from their respective full-length human cDNA. All these MON1A, CCZ1, C18orf8, Rab7A full-length or fragments were cloned into in-house modified versions of the pALink vector or pET32a vector for recombinant protein expressions. Meanwhile, for the co-immunoprecipitation assays, the full-length or fragments of MON1A, CCZ1, C18orf8 and Rab7A were cloned into pA-Flag, pA-Myc, pA-HA, and pEGFP-C1 vectors, respectively. All the single or multiple point mutations of MON1A, CCZ1, C18orf8 and Rab7A used in this study were created using the standard polymerase chain reaction (PCR)-based mutagenesis method, and further confirmed by DNA sequencing.

Protein expression and purification

The MON1A(235-652), 3×Flag-tagged CCZ1(1-482) and C18orf8(1-657) proteins were co-expressed in HEK293F cells using transient transfection method. The cells were grown at 37 °C in Union-293 medium (Union-Biotech, UP1000). On the day before transfection, the cells were seeded to the density of 1×10^6 cells/mL. Next, 600 mL cells at density of 2×10^6 cells/mL were transfected by adding the transfection mixture on the day of transfection. The transfection mixture consists of four components: 240 μg of pAlink MON1A(235-652) plasmid, 240 μg pAlink-3×Flag CCZ1(1-482) plasmid and 240 μg of pAlink C18orf8(1-657), which were then pre-diluted in 24 mL of Union-293 medium and

mixed with 1.8 mg Polyethylenimine 40K (PEI 40K, Polysciences CatNo 23966-1). About 48-72 hours after transfection, the cells were enriched by centrifugation at 3,000 rpm for 10 minutes at 4 °C. Then, cells were lysed using a lysis buffer containing 50 mM Tris-HCl, 200 mM NaCl, 2 mM $MgCl_2$, 10% glycerol, 1% TritonX-100, 1 μg/mL Aprotinin, 0.5 μg/mL Pepstatin, 0.5 μg/mL Leupeptin and 0.5 mM PMSF. Lysates were cleared by ultracentrifugation at 14,000 rpm for 30 minutes at 4 °C. Subsequently, the supernatant was incubated with 800 μL anti-DYKDDDDK G1 Affinity Resin (GenScript) for 60 minutes at 4 °C. Beads were washed with the wash buffer (20 mM Tris, pH 7.5, 200 mM NaCl, 1 mM DTT and 5% glycerol) after incubation. Finally, beads were eluted with 1 mL 3×Flag peptide (0.5 mg/mL) for 60 minutes at 4 °C, for four times with a total of 4 mL peptides. The supernatant was gathered and filtered through a 0.22 μm syringe filter, and then concentrated to about 0.5 mL for further purification by size-exclusion chromatography equilibrated with a column buffer containing 20 mM Tris, 100 mM NaCl, 1 mM DTT at pH 7.5.

As for GST-tagged Rab7A(1-181), it was expressed in BL21 (DE3) *E. coli* cells induced by 150 μM isopropyl-β-D-thiogalactopyranoside at 16 °C. The bacterial cell pellets were re-suspended in the binding buffer (20 mM Tris, 100 mM NaCl, 1 mM DTT at pH 7.5), and then lysed by the ultrahigh-pressure FB-110XNANO homogenizer machine (Shanghai Litu Machinery Equipment Engineering Co., Ltd.). Then, the Lysates were cleared by ultracentrifugation at 17,000 rpm (35,000 g) for 30 minutes at 4 °C to remove the debris. Subsequently, the GST-tagged Rab7A(1-181) was initially purified by glutathione sepharose 4B (GE Healthcare) affinity chromatography. Proteins were further purified by size-exclusion chromatography equilibrated with a column buffer containing

20 mM Tris, 100 mM NaCl, 1 mM DTT at pH 7.5. The N-terminal GST tags of relevant recombinant proteins were cleaved by 3C protease and further removed by size-exclusion chromatography or glutathione sepharose 4B (GE Healthcare) affinity chromatography.

Size exclusion chromatography

Size exclusion chromatography assays were carried out on an AKTA FPLC system (GE Healthcare). The MON1A/CCZ1/C18orf8 complex protein and Rab7A WT or mutants protein samples (500 μl) were loaded on to a Superdex^{TM} 200 Increase 10/300 GL column (GE Healthcare), and eluted in a buffer containing 20 mM Tris, 100 mM NaCl, 1 mM DTT at pH 7.5 with monitoring the absorbance at 280 nm by the AKTA FPLC system (GE Healthcare). The results were further fitted through Origin 9 software and aligned with each other.

Multi-angle light scattering analysis

For MALS measurement, MON1A/CCZ1/C18orf8/Rab7A T22N complex protein were injected into an AKTA FPLC system (GE Healthcare) with a Superdex 200 Increase 10/300 column connected to the detectors (DAWN HELEOS II and Optilab T-rEX, WYATT). Proteins were eluted out in the buffer containing 20 mM Tris, 100 mM NaCl, 1 mM DTT at pH 7.5 at the flow rate of 0.5 mL/min. The chromatography system was coupled to a static light scattering detector (miniDawn, Wyatt Technology) and a differential refractive index detector (Optilab, Wyatt Technology). Data were collected every 0.5 s with a flow rate of 0.5 mL/min. Data were analyzed using the ASTRA 6 software (Wyatt Technology) and drawn using the Origin 9 software.

Cryo-EM sample preparation and data collection

The MON1A/CCZ1/C18orf8/Rab7A (MCCR) complex (0.5 mg/mL) solutions were applied to freshly glow-discharged 200 mesh 2/1 Au grids (Quantifoil), respectively. The grids were prepared using a Vitrobot Mark IV (Thermo-Fisher) at 4 °C under 100% humidity conditions, with a waiting time of 10 s, a blot time of 2 s, and a blot force of -2. The grids were plunged into cooled liquid ethane.

The grids containing the MCCR complex were loaded into a 300 kV Titan Krios G4 microscope (Thermo-Fisher) equipped with a Biocontinuum K3 Direct Electron Detector and a Gatan GIF imaging filter. Data collection was performed at a magnification of 81,000x, corresponding to a pixel size of 0.5275 Å per pixel in super-resolution mode. For the MCCR complex, each movie was dose-fractionated into 40 frames and recorded using a total dose of 49.41 electrons per $Å^2$. The data were automatically acquired using the image-shift method in the EPU software (Deng et al., 2021) with a defocus range of -1.4 to -2.4 μm.

Cryo-EM data processing and model building

The movies were imported into RELION-3.1 (Zivanov et al., 2018), motion-corrected, and electron-dose-weighted with MotionCorr2 (Zheng et al., 2017). The CTF was estimated using CTFFIND4 (Rohou and Grigorieff, 2015). The particles were first picked with Gautomatch (https://www2.mrc-lmb.cam.ac.uk/download/gautomatch-053/), extracted from micrographs, and subjected to 2D classification and CryoSPARC (Punjani

et al., 2017) ab-initio reconstruction for 2D and 3D templates. The resulting 2D averages were then used as templates to pick the dataset again with Gautomatch.

For the MCCR complex, a total of 545,235 particles were picked using Gautomatch with templates were extracted from the micrographs and subjected to multiple rounds of heterogeneous refinement in CryoSPARC to remove incomplete complexes and classes with preferred orientation. The 77,193 particles from the best class were then subjected to non-uniform refinement in CryoSPARC, generating a 3.41 Å resolution map.

We used the AlphaFold2 (Jumper et al., 2021) predicted structures for human MON1A, CCZ1, C18orf8, and Rab7A as initial models. These models were first fitted as rigid bodies into the cryo-EM density maps using UCSF Chimera (Pettersen et al., 2004) and then manually adjusted in Coot (Emsley and Cowtan, 2004). The adjusted models were subjected to iterative rounds of real-space refinement in PHENIX (Adams et al., 2002).

Cell culture, transfection and co-immunoprecipitation assay

HEK293T cells were cultured in Dulbecco's modified Eagle's medium (DMEM, Invitrogen) supplemented with 10% fetal bovine serum (FBS, Invitrogen). Flag-tagged MON1A, Myc-tagged CCZ1, HA-tagged C18orf8 and Enhanced GFP (EGFP)-tagged Rab7A plasmids (WT, mutants, full-length or fragments) were co-transfected into HEK293T cells using Lipofectamine 2000 transfection reagent (Thermo Fisher Scientific). Cells were collected 36 hours after transfection and lysed in ice-cold cell lysis buffer containing 50 mM Tris-HCl, 150 mM NaCl, 0.5% NP-40, 1 mM PMSF, and 1% protease inhibitor cocktail at pH 7.5 for 40 minutes at 4 °C. Lysates were centrifuged at

14,500 g for 20 minutes at 4 °C to separate soluble fractions and cell debris. Supernatants were applied to anti-Flag M2 Affinity Gel (Sigma, product number: A2220) and incubated for 15 minutes at 4 °C. The beads and non-bound proteins were separated by centrifugation at 800 g for 3 minutes at 4 °C. After washing three to five times with the cold wash buffer (50 mM Tris-HCl, 150 mM NaCl, and 0.05% NP-40 at pH 7.5), the beads were resuspended with the SDS-PAGE sample buffer and boiled for 10 minutes at 100 °C. The prepared samples were analyzed by SDS-PAGE. The Flag-tagged MON1A, Myc-tagged CCZ1, HA-tagged C18orf8 and EGFP-tagged Rab7A were detected by Western blot using the Flag antibody (1:2000 dilution; proteintech, catalog no. 20543-1-AP), Myc antibody (1:4000 dilution; proteintech, catalog no. 16286-1-AP), HA antibody (1:4000 dilution; proteintech, catalog no. 51064-2-AP), and GFP antibody (1:1000 dilution; proteintech, catalog no. 50430-2-AP), respectively.

References

Adams, P.D., Grosse-Kunstleve, R.W., Hung, L.-W., Ioerger, T.R., McCoy, A.J., Moriarty, N.W., Read, R.J., Sacchettini, J.C., Sauter, N.K., and Terwilliger, T.C. (2002). PHENIX: building new software for automated crystallographic structure determination. Acta Crystallographica Section D 58, 1948-1954.

Deng, Y., Grollios, F., Kohr, H., van Knippenberg, B., Janus, M., and Caglar, F. (2021). Smart EPU: SPA Getting Intelligent. Microscopy and Microanalysis 27, 454-455.

Emsley, P., and Cowtan, K. (2004). Coot: model-building tools for molecular graphics. Acta Crystallogr D Biol Crystallogr 60, 2126-2132.

Jumper, J., Evans, R., Pritzel, A., Green, T., Figurnov, M., Ronneberger, O., Tunyasuvunakool, K., Bates, R., Zidek, A., Potapenko, A., *et al.* (2021). Highly accurate protein structure prediction with AlphaFold. Nature 596, 583-589.

Pettersen, E.F., Goddard, T.D., Huang, C.C., Couch, G.S., Greenblatt, D.M., Meng, E.C., and Ferrin, T.E. (2004). UCSF Chimera--a visualization system for exploratory research and analysis. J Comput Chem 25, 1605-1612.

Punjani, A., Rubinstein, J.L., Fleet, D.J., and Brubaker, M.A. (2017). cryoSPARC: algorithms for rapid unsupervised cryo-EM structure determination. Nature Methods 14, 290-296.

Rohou, A., and Grigorieff, N. (2015). CTFFIND4: Fast and accurate defocus estimation from electron micrographs. Journal of Structural Biology 192, 216-221.

Zheng, S.Q., Palovcak, E., Armache, J.-P., Verba, K.A., Cheng, Y., and Agard, D.A. (2017). MotionCor2: anisotropic correction of beam-induced motion for improved cryo-electron microscopy. Nature Methods 14, 331-332.

Zivanov, J., Nakane, T., Forsberg, B.O., Kimanius, D., Hagen, W.J.H., Lindahl, E., and Scheres, S.H.W. (2018). New tools for automated high-resolution cryo-EM structure determination in RELION-3. eLife 7, e42166.

Supplemental figures:

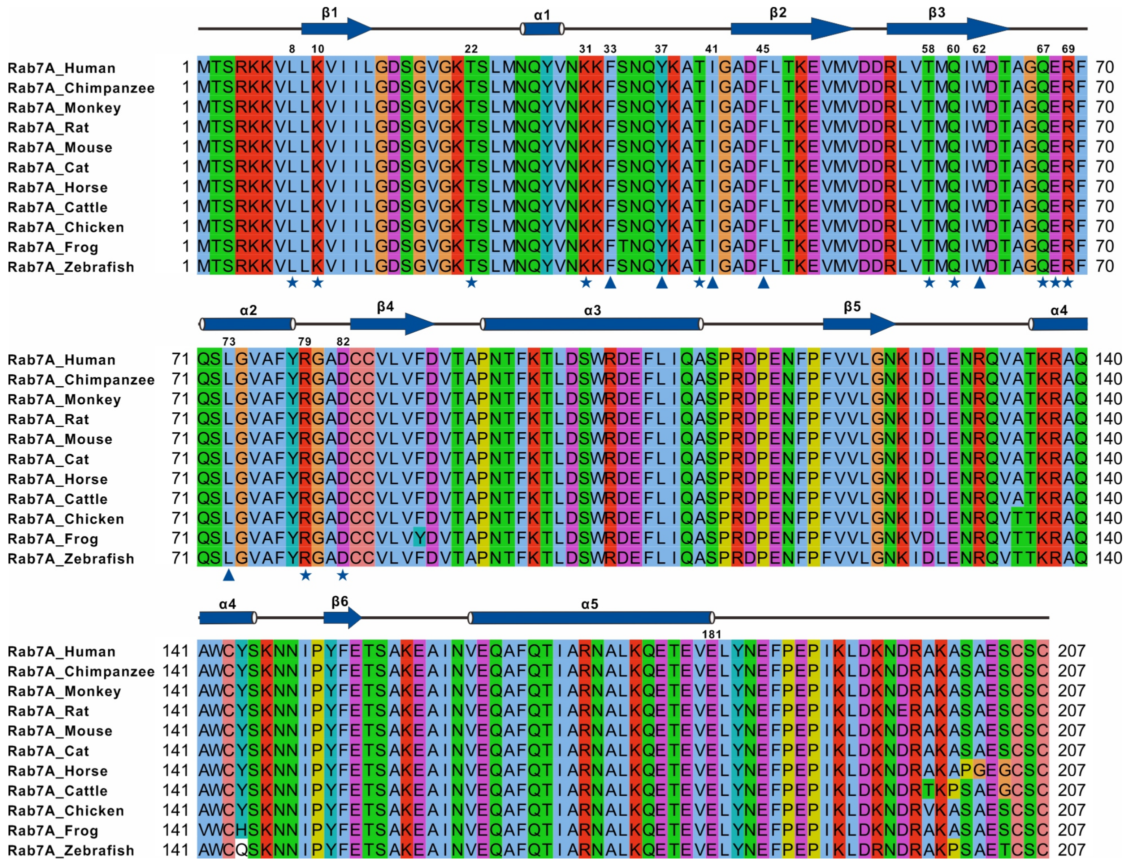

Fig. S1. Structure-based sequence alignment analysis of Rab7A from different species. In this alignment, the conserved residues are highlighted by colors using software Jalview2.8.1 (http://www.jalview.org/). The binding interface residues of Rab7A, which are important for the interaction with the MON1A/CCZ1 sub-complex, are highlighted with marine stars (polar interactions) or marine triangles (hydrophobic interactions).

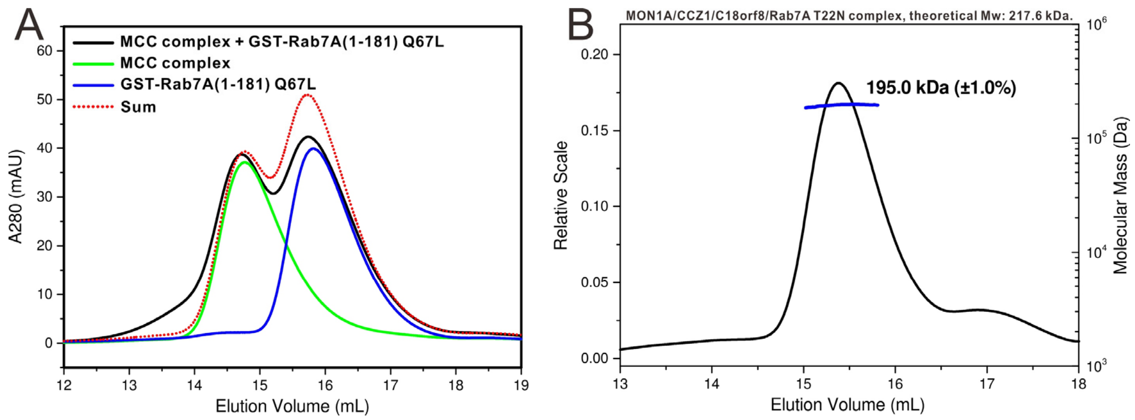

Fig. S2. Biochemical characterizations of the interaction between the MON1A/CCZ1/C18orf8 complex and Rab7A. (**A**) Size exclusion chromatography (SEC)-based analyses of the interaction between the MON1A/CCZ1/C18orf8 complex and the GTP-bound Rab7A(1-181) Q67L mutant. A280, the UV absorbance at 280 nm. (**B**) The plot of the MALS result of the MON1A/CCZ1/C18orf8/Rab7A T22N complex shows the relative light scattering signal as a function of elution volume. The measured molecular weight is shown in blue, and the molecular weight error is obtained from the data analysis software (ASTRA) and showed in the bracket.

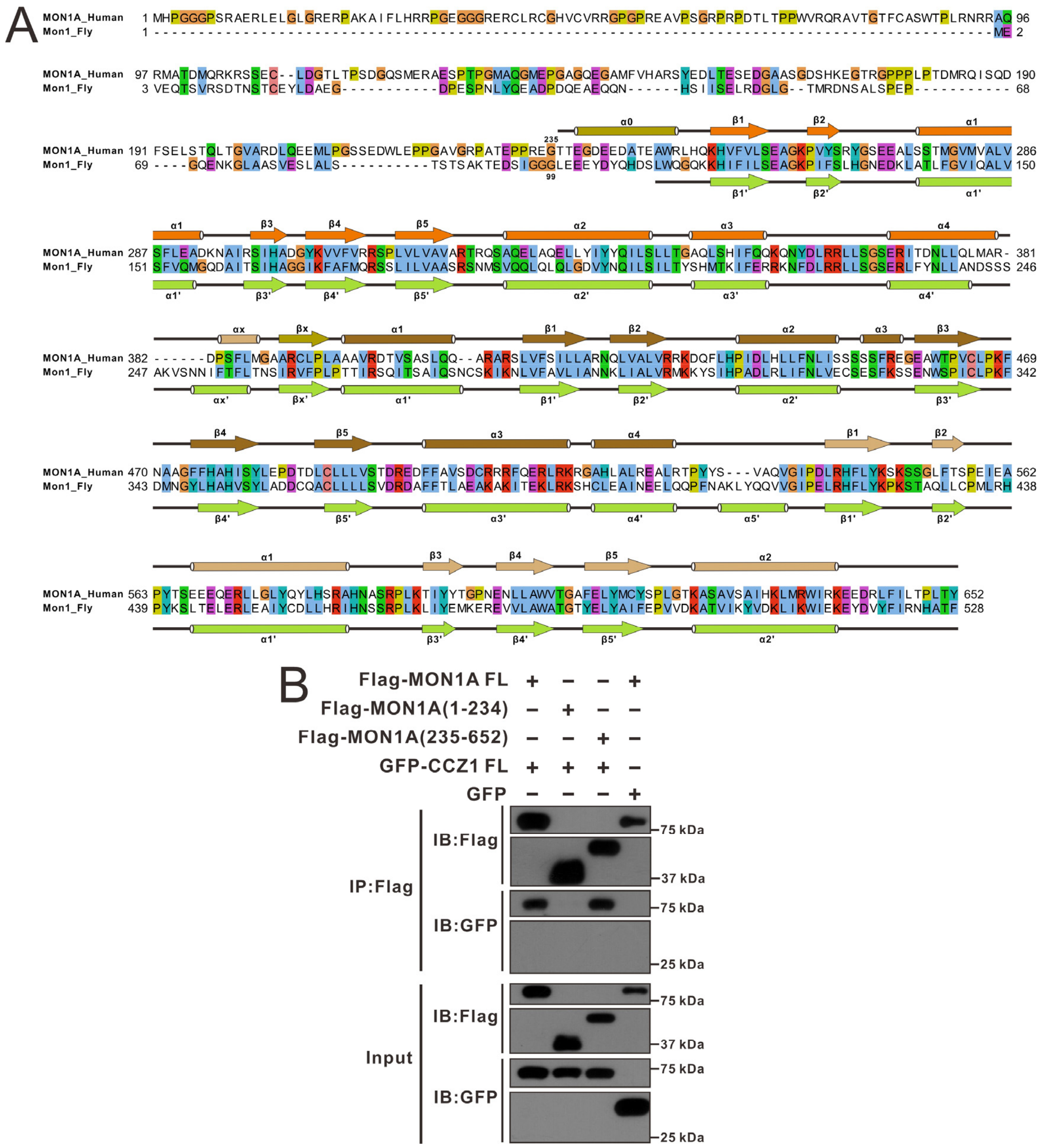

Fig. S3. The rationale for using the truncated MON1A(235-652) to assemble the MON1A/CCZ1/C18orf8 complex. (**A**) Structure-based sequence alignment analysis of human MON1A with Drosophila Mon1. In this alignment, the conserved residues are highlighted by colors using software Jalview2.8.1 (http://www.jalview.org/). The secondary structures of human MON1A LD1, MON1A LD2, MON1A LD3 and Drosophila Mon1 are colored in bright orange, light orange, orange and limon,

respectively. (**B**) Co-immunoprecipitation assays showing that the full-length MON1A and the C-terminal MON1A(235-652) fragment can well interact with CCZ1 in cells, while the N-terminal MON1A(1-234) fragment is unable to interact with CCZ1. "IB" stands for immunoblotting.

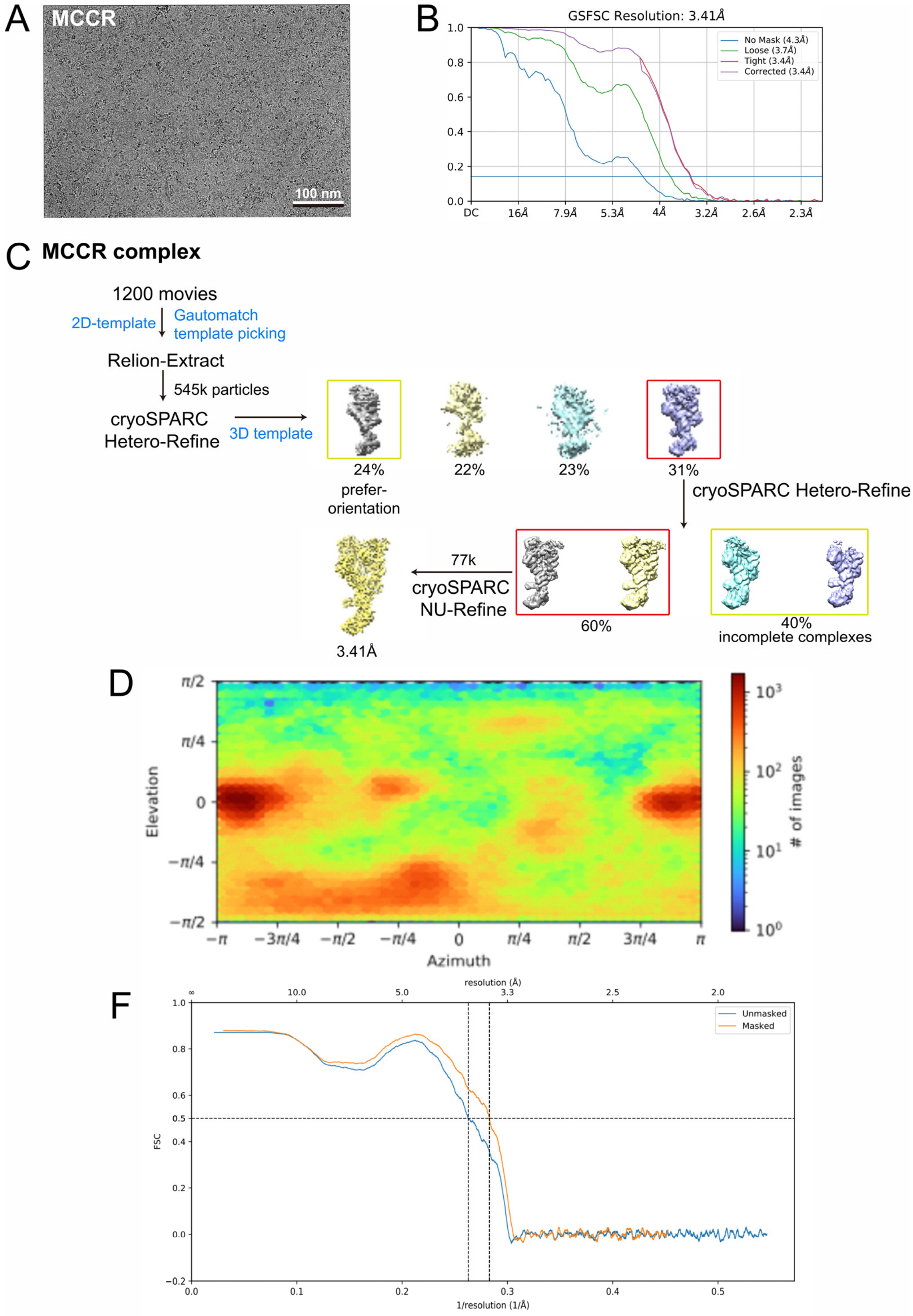

Fig. S4. Cryo-EM data processing of the MON1A/CCZ1/C18orf8/Rab7A complex.

(**A**) Representative motion-corrected electron micrograph of the

MON1A/CCZ1/C18orf8/Rab7A (MCCR) complex. Scale bar, 100 nm. (**B**) Gold-standard FSC curves (FSC = 0.143) of the MON1A/CCZ1/C18orf8/Rab7A complex. (**C**) Image-processing workflow of the MON1A/CCZ1/C18orf8/Rab7A complex using RELION and cryoSPARC. See Materials and methods as well as Table S1 for details. (**D**) Orientation distribution plot. Orientation distribution of the particles used in the final reconstruction of the MON1A/CCZ1/C18orf8/Rab7A complex calculated in cryoSPARC. (**E**) Map versus model FSC curves with and without mask calculated in Phenix.

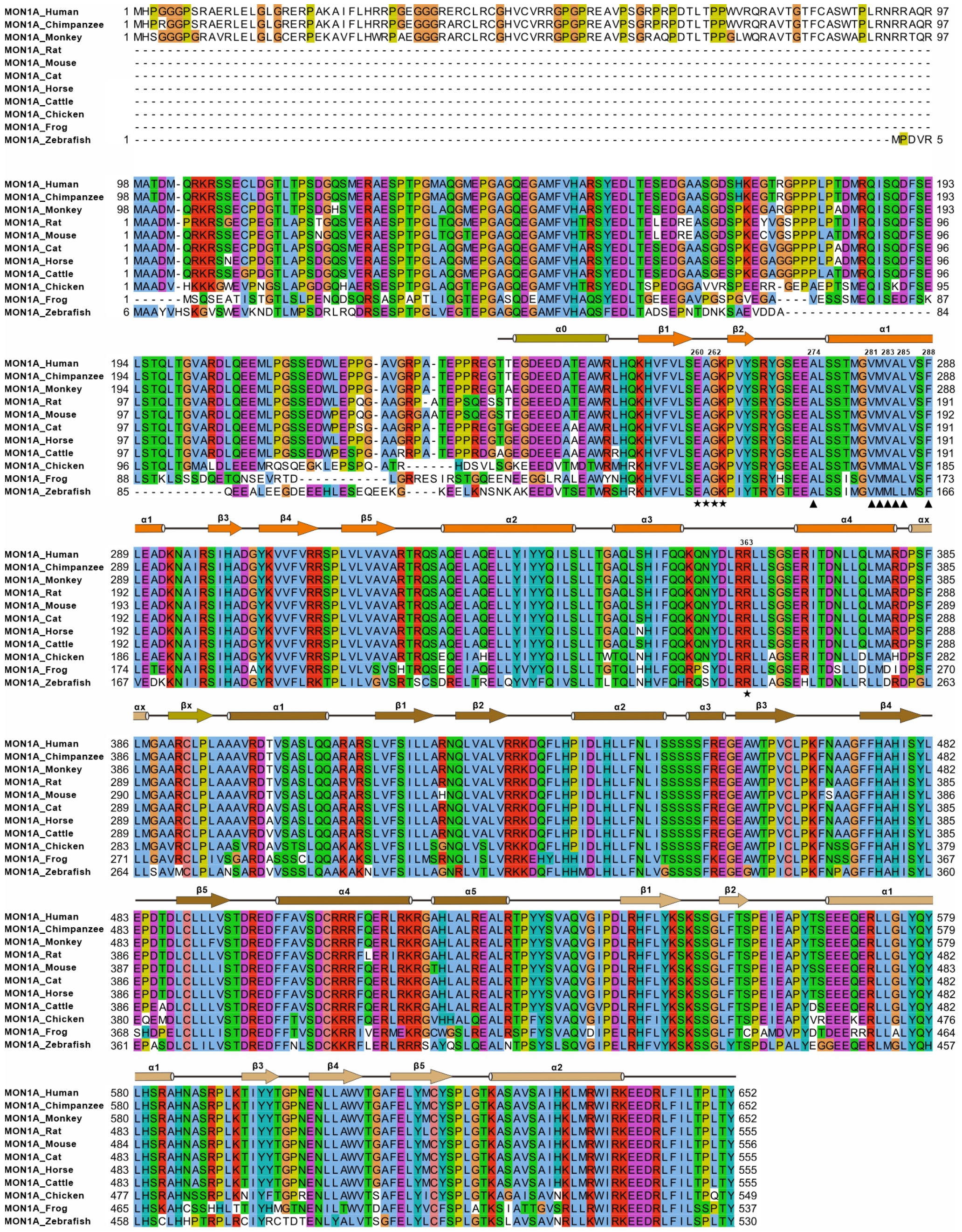

Fig. S5. Structure-based sequence alignment analysis of MON1A from different species. In this alignment, the conserved residues are highlighted by colors using software Jalview2.8.1 (http://www.jalview.org/). The binding interface residues of MON1A, which are important for the interactions with Rab7A are highlighted with black stars (polar interactions) or black triangles (hydrophobic interactions).

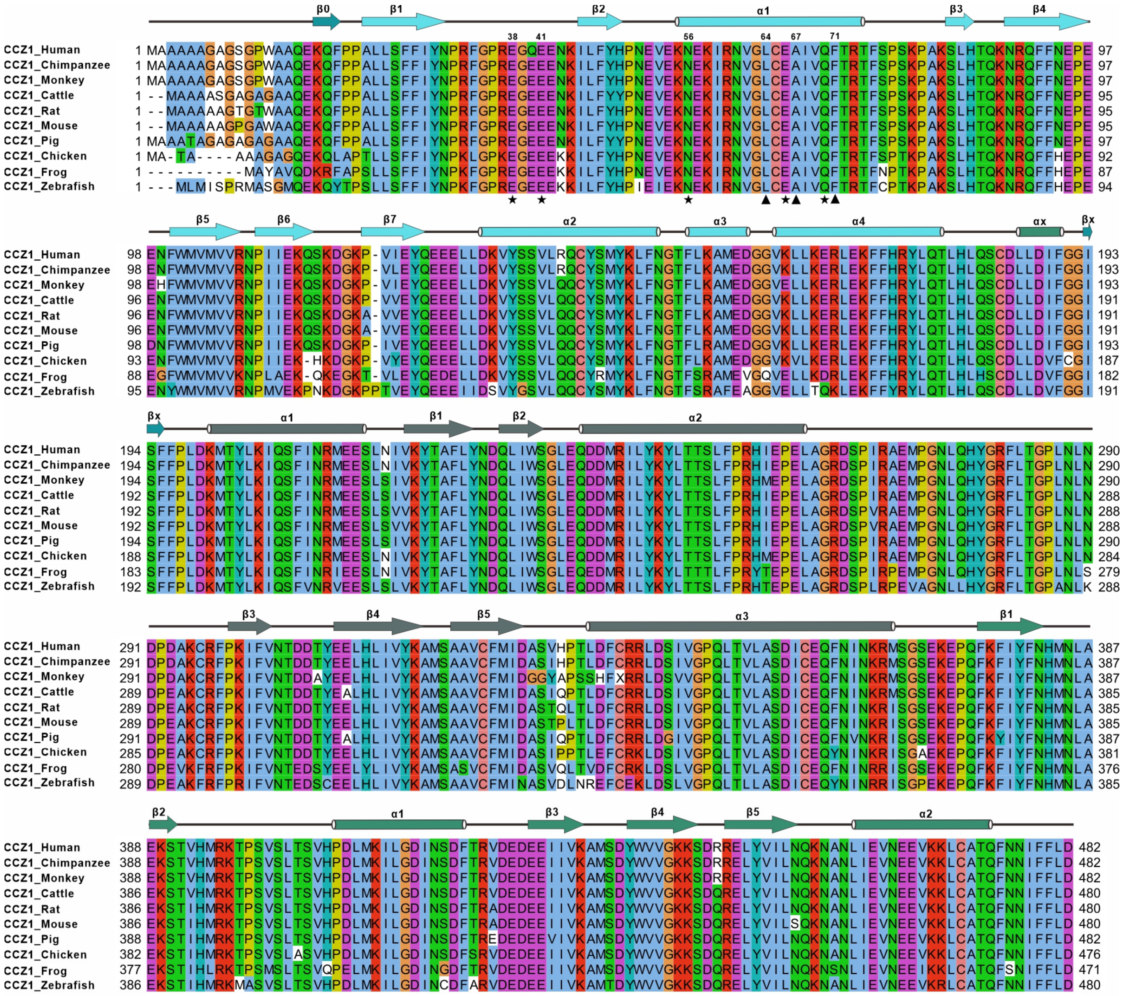

Fig. S6. Structure-based sequence alignment analysis of CCZ1 from different species. In this alignment, the conserved residues are highlighted by colors using software Jalview2.8.1 (http://www.jalview.org/). The binding interface residues of CCZ1, which are important for the interactions with Rab7A are highlighted with black stars (polar interactions) or black triangles (hydrophobic interactions).

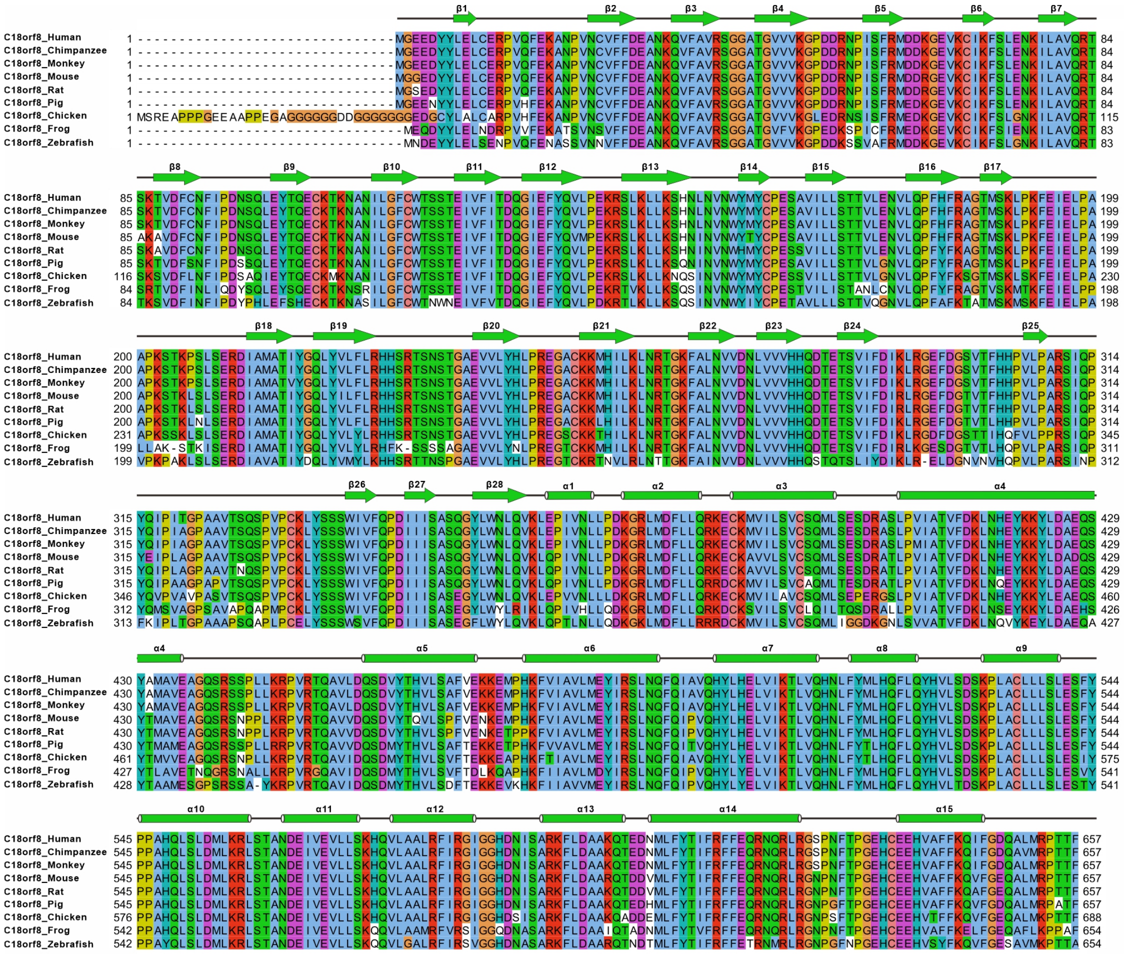

Fig. S7. Structure-based sequence alignment analysis of C18orf8 from different species. In this alignment, the conserved residues are highlighted by colors using software Jalview2.8.1 (http://www.jalview.org/).

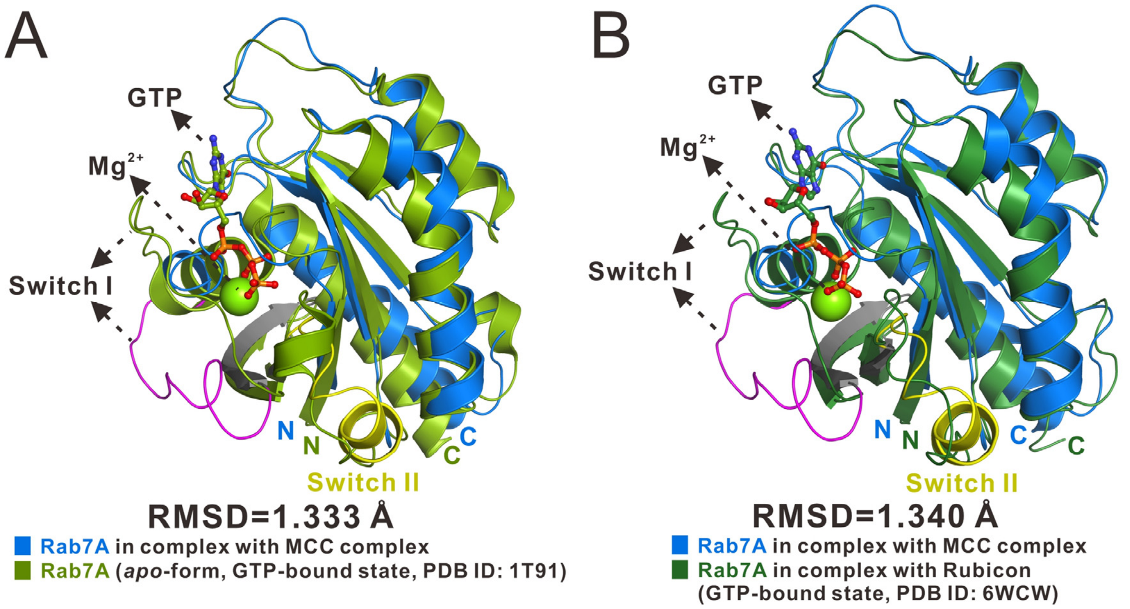

Fig. S8. Structural analyses of the MON1A/CCZ1/C18orf8/Rab7A complex. (**A** and **B**) Ribbon diagram showing the structural comparison of the Rab7A in the MON1A/CCZ1/C18orf8/Rab7A complex with the *apo*-form GTP-bound active Rab7A (PDB ID: 1T91) (**A**), or the GTP-bound active Rab7A in the Rubicon/Rab7A complex (PDB ID: 6WCW) (**B**).

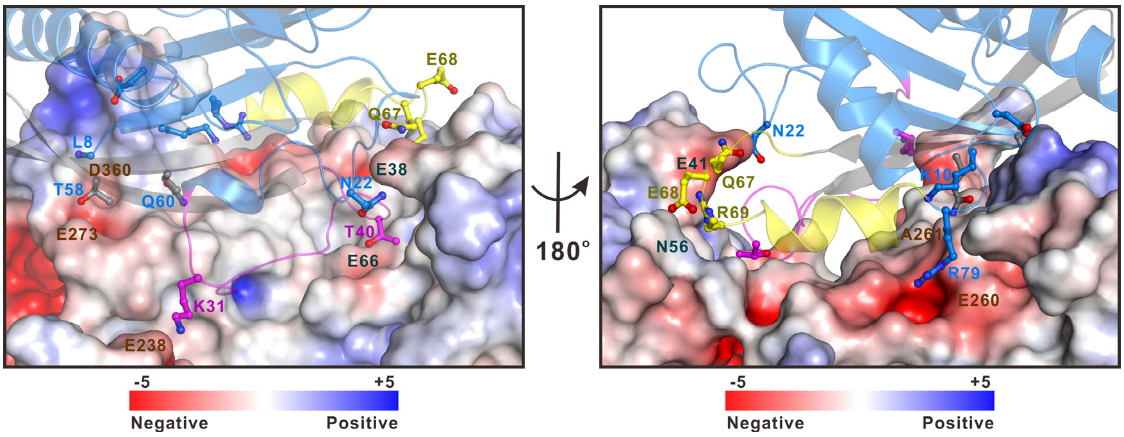

Fig. S9. Structural analysis of the binding interface between the MON1A/CCZ1/C18orf8 complex and Rab7A. The combined surface charge potential representation and the ribbon-stick model showing the detailed interactions between the MON1A/CCZ1/C18orf8 complex and Rab7A.

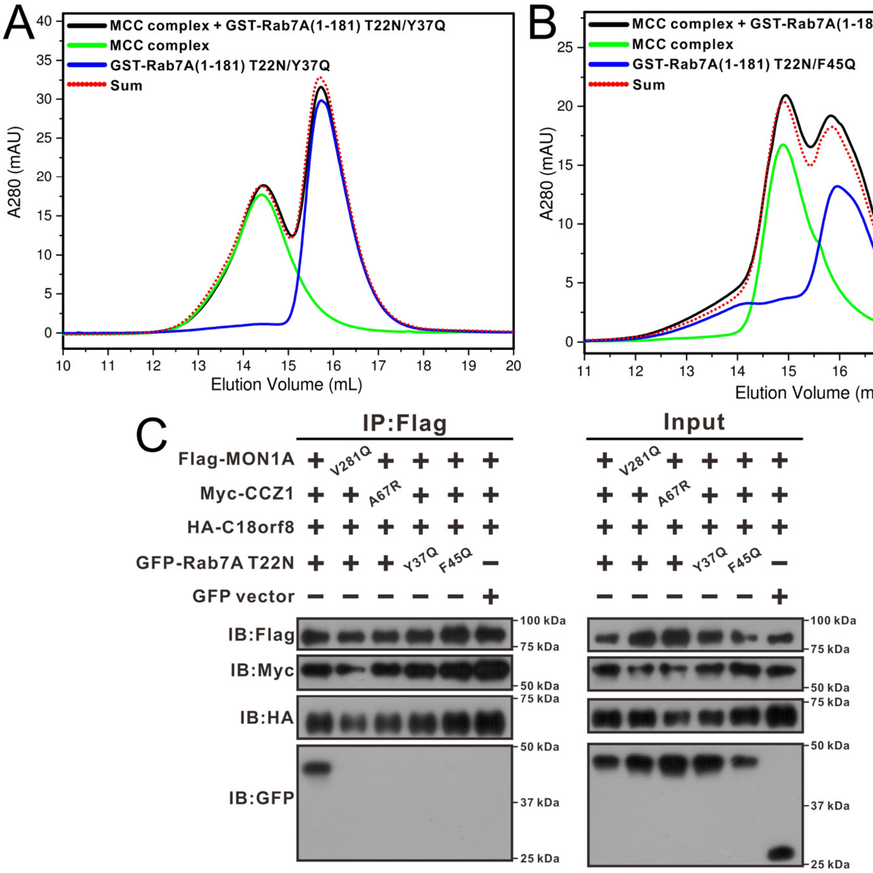

Fig. S10. The biochemical validation of the binding interface between the MON1A/CCZ1/C18orf8 complex and Rab7A. (**A** and **B**) SEC-based analyses of the interaction of the MON1A/CCZ1/C18orf8 complex with the Rab7A(1-181) T22N/Y37Q mutant (**A**), or the Rab7A(1-181) T22N/F45Q mutant (**B**). A280, the UV absorbance at 280 nm. (**C**) Co-immunoprecipitation assays showing that point mutations of key binding interface residues of MON1A, CCZ1 or Rab7A observed in the MON1A/CCZ1/C18orf8/Rab7A complex structure essentially disrupt their specific interaction in cells. "IB" stands for immunoblotting.

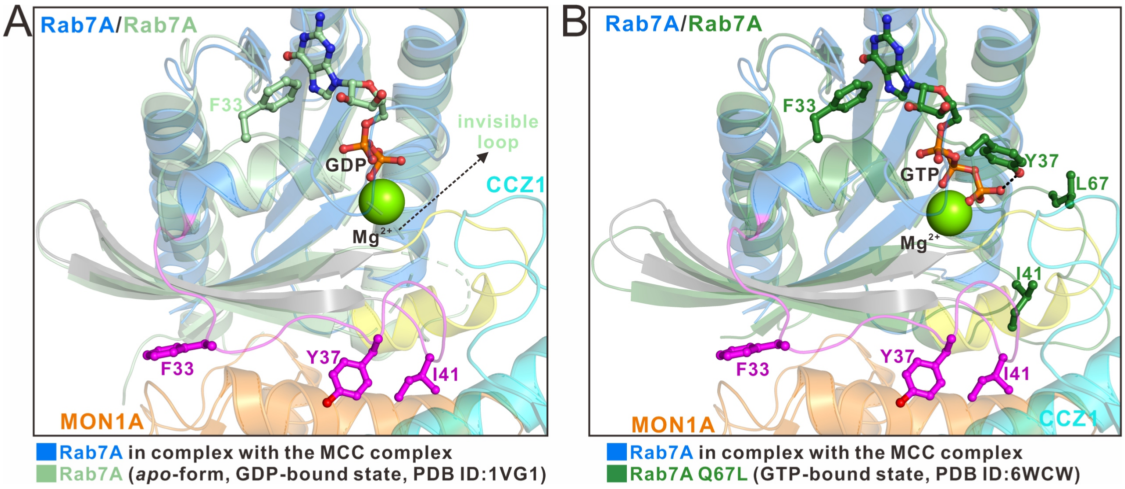

Fig. S11. Structural comparison analyses of Rab7A in the MON1A/CCZ1/C18orf8/Rab7A complex with different forms of Rab7A. (**A** and **B**) The ribbon-stick representation showing the detailed structure comparison of Rab7A in the MON1A/CCZ1/C18orf8/Rab7A complex with the GDP-bound wild-type Rab7A (PDB ID: 1VG1) (**A**), or the GTP-bound Rab7A Q67L mutant in the Rubicon RH/Rab7A Q67L complex (PDB ID: 6WCW) (**B**). In these drawings, the side chains of the key residues of Rab7A as well as the bound GDP, GTP molecules in Rab7A are shown in the stick-ball mode, and the related hydrogen bond between the side chain hydroxyl group of Rab7A Y37 and GTP is shown as a dotted line. Notably, the side chain hydroxyl group of Y37 in the GTP-bound Rab7A forms a strong hydrogen bond with the γ-phosphate group of the GTP molecule bound with Rab7A in panel **B**.

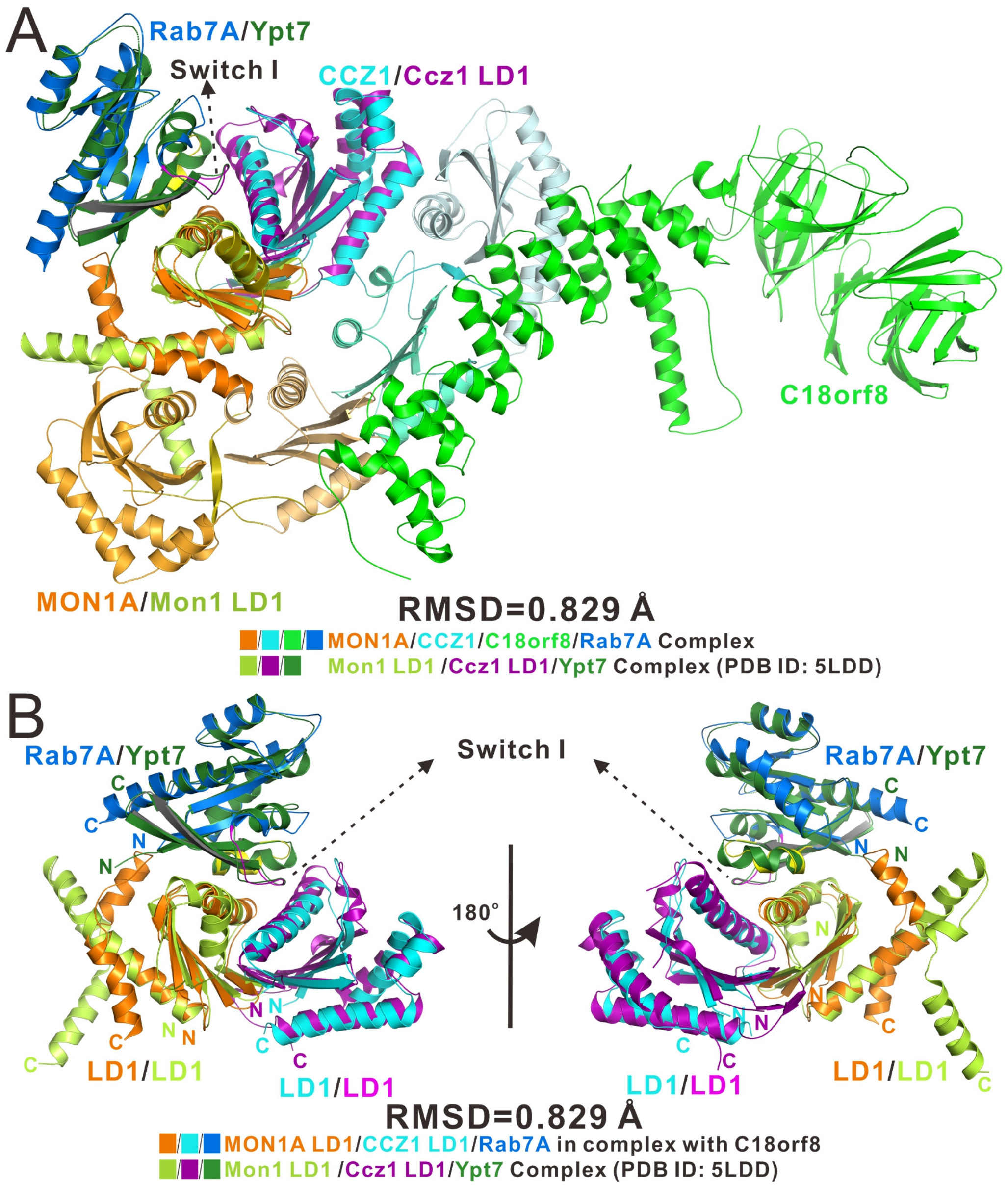

Fig. S12. Structural comparison analyses of the MON1A/CCZ1/C18orf8/Rab7A complex with the fungal Mon1 LD1/Ccz1 LD1/Ypt7 complex. (**A**) Ribbon diagram showing the structural comparison of the human MON1A/CCZ1/C18orf8/Rab7A complex with the fungal Mon1 LD1/Ccz1 LD1/Ypt7 complex (PDB ID: 5LDD). (**B**) Ribbon diagram showing the structural comparison of the human MON1A LD1/CCZ1 LD1/Rab7A complex in the MON1A/CCZ1/C18orf8/Rab7A complex with the fungal Mon1 LD1/Ccz1 LD1/Ypt7 complex (PDB ID: 5LDD).

Supplemental Table

Table S1: Cryo-EM Data collection, refinement and validation statistics.

	MON1A/CCZ1/C18orf8/Rab7A T22N complex (EMD-62697) (PDB-9L0D)
**Data collection and processing**	
Magnification	81,000
Voltage (kV)	300
Electron exposure (e–/Å^2)	49.41
Defocus range (μm)	-1.4 ~ -2.4
Pixel size (Å)	1.055
Symmetry imposed	C1
Initial particle images (no.)	545,235
Final particle images (no.)	77,193
Map resolution (Å)	3.41
FSC threshold	0.143
Map resolution range (Å)	2.5-5.5
**Refinement**	
Initial model used (PDB code)	predicted
Map sharpening *B* factor (Å^2)	-107.0
Model composition	
Non-hydrogen atoms	13894
Protein residues	1718
Ligands	0
R.m.s. deviations	
Bond lengths (Å)	0.007
Bond angles (°)	0.843
Validation	
MolProbity score	2.85
Clash score	16.67
Poor rotamers (%)	0
Ramachandran plot	
Outliers (%)	0
Allowed (%)	7.02
Favored (%)	92.98
